# Why PEDOT:PSS Should Not Be Used for Raman Sensing of Redox States (and How It Could Be)

**DOI:** 10.1021/acsami.2c17147

**Published:** 2022-12-07

**Authors:** Ivano Alessandri, Fabrizio Torricelli, Beatrice Cerea, Michele Speziani, Paolo Romele, Zsolt Miklos Kovacs-Vajna, Irene Vassalini

**Affiliations:** † Department of Information Engineering, University of Brescia, via Branze 38, 25123Brescia, Italy; ‡ INSTM-National Consortium for Materials Science and Technology, UdR Brescia, via Branze 38, 25123Brescia, Italy; § CNR-INO, UdR Brescia, via Branze 38, 25123Brescia, Italy

**Keywords:** PEDOT:PSS, Raman, electrolyte, ion diffusion dynamics, charge transfer, saline buffers, optical sensors

## Abstract

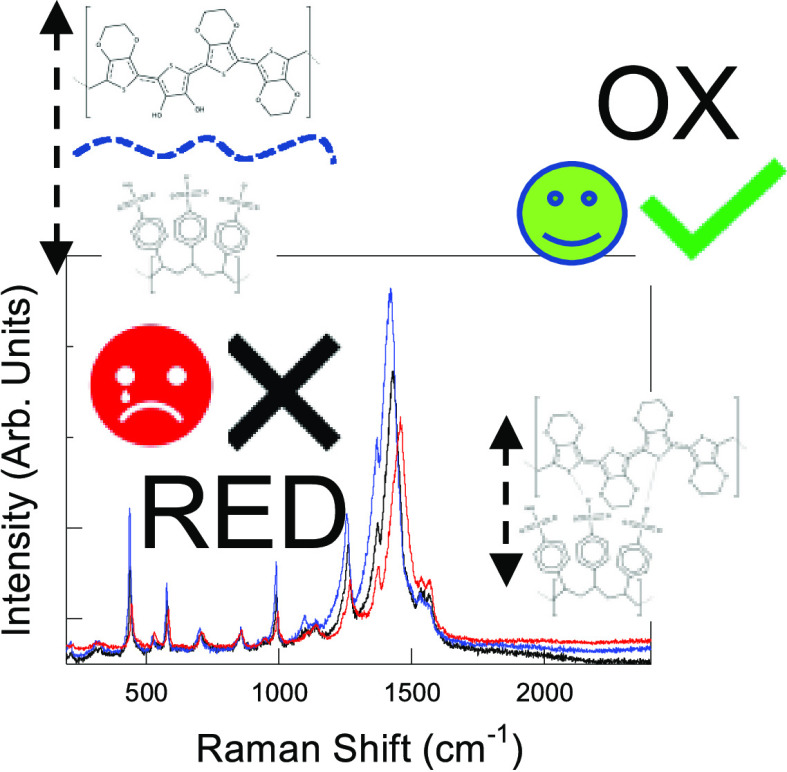

Poly(3,4-ethylenedioxythiophene):poly(styrene sulfonate) (PEDOT:PSS) has been recently proposed for Raman sensing of redox-active species in solution. Here, we investigated the rationale of this approach through systematic experiments, in which the Raman spectrum of PEDOT:PSS was analyzed in the presence of either nonoxidizing or oxidizing electrolytes. The results demonstrated that Raman spectra precisely reflect the conformation of PEDOT units and their interactions with PSS. Two different responses were observed. In the case of oxidizing electrolytes, the effect of charge transfer is accurately transduced in Raman spectrum changes. On the other hand, reduction induces a progressive separation between the PEDOT and PSS chains, which decreases their mutual interaction. This stimulus determines characteristic variations in the intensity, shape, and position of the Raman spectra. However, we demonstrated that the same effects can be obtained either by increasing the concentration of nonoxidizing electrolytes or by deprotonating PSS chains. This poses severe limitations to the use of PEDOT:PSS for this type of Raman sensing. This study allows us to revise most of the Raman results reported in the literature with a clear model, setting a new basis for investigating the dynamics of mixed electronic/ionic charge transfer in conductive polymers.

## 1 Introduction

Poly(3,4-ethylenedioxythiophene):poly(styrene sulfonate) (PEDOT:PSS) is one of the most versatile materials for large-area electronics and bioelectronics with a variety of application in flexible devices,^1^ organic solar cells,^2^ sensors,^3^ thermoelectrics,^4^ and organic electrochemical transistors (OECTs).^5,6^ In addition, recently developed strategies improved its biodegradability, making PEDOT:PSS a promising candidate for green electronics.^7−9^ PEDOT:PSS is a mixture of two ionomers, PEDOT, which is conductive, and PSS, which is insulating and normally added in excess to make PEDOT soluble in water and suitable for processing in thin-film fabrication, as well as for stabilizing its oxidation state. Unlike most of other conductive polymers, pristine PEDOT is p-doped since its synthesis proceeds through the oxidative polymerization of 3,4-ethylenedioxythiophene (EDOT) monomers, achieved by means of the concurrent reduction of Fe^3+^-based salts. The maximum oxidation level of the PEDOT chains is around 33%, which corresponds to one charge per three monomer units.^10^ The oxidation state of the PEDOT units can be directly inferred from their optical properties. Neutral PEDOT shows an intense, broad optical absorption band centered around 610 nm, while oxidation shifts the absorption in the near-infrared. This effect has been exploited for fabricating electrochromic devices that reversibly change their color as a function of doping/dedoping.^11^ This modulation of optical absorption is also at the basis of the use of Raman spectroscopy for characterizing PEDOT and monitoring the variation of its electronic state as a function of the applied voltage.^12,13^ In recent years, there has been an intense research activity aimed at exploiting the Raman signal of PEDOT:PSS to perform enzymeless/contactless sensing of redox-active molecules and for the spectroscopic determination of the redox potential of biological solutions and fluids.^13,14^ This approach assumes that the information on the redox state of an analyte can be accurately transduced through the modification of the oxidation state of PEDOT, which should generate a specific modification of the Raman signals associated to thiophene moieties. However, that hypothesis does not account for complexity originated from the mixed electronic/ionic nature of charge transport in three-dimensional PEDOT:PSS networks in electrolytic media, giving rise to misinterpretations of the experimental data.

Here, we report an extensive study of these processes through a series of experiments that investigate the Raman response of PEDOT:PSS films in different types of electrolytic environments. The results reveal that, in the case of interaction with nonoxidizing species, the same type of Raman response can be induced by different factors, not necessarily related to the redox state of a target analyte. This makes Raman sensing not applicable for achieving quantitative, reliable data in real working conditions. In parallel, this study demonstrates the high sensitivity of Raman scattering toward the structural conformation of PEDOT:PSS and mutual interactions between the chains of the two ionomers, which opens exciting perspectives for a precise characterization of ionic strength and diffusion dynamics of electrolytes through the polymeric network.

## 2 Experimental Section

All of the PEDOT:PSS films utilized in this work were fabricated from commercial PEDOT:PSS (Clevios PH-500, PEDOT:PSS ratio: 1:2.5 by weight, pH = 2.5 at 20 °C, density: 1 g cm^–3^ at 20 °C). The PEDOT:PSS solution was formulated with ethylene glycol (EG, 5% v/v of the PEDOT:PSS solution) and 3-glycidoxypropyltrimethoxysilane (GOPS, 1% v/v of the PEDOT:PSS solution) and drop-casted on microscope glass slides previously treated with acetone, ethanol, water, and ozone–UV cleaning. All types of samples were cured at 140 °C for 15 min to promote cross-linking and increase their mechanical stability and adhesiveness to the glass support. In the case of OECTs, the complete protocol of fabrication is reported in ref (15). Details on the experiments with OECTs are reported in Supporting Information Sections S1 and S2.

The Raman experiments were carried out by soaking the glass-supported films into aqueous solutions containing different types of analytes/electrolytes for 30 min. The analytes included l-glutathione (from 1 to 10 mM), while ionic electrolytes included FeNO_3_ (10 and 100 mM), halide salts (NaCl, KBr, from 10 to 100 mM), and saline buffers such as PBS (1 and 10×) and other buffers for pH standardization (potassium hydrogen phthalate 0.2 M, potassium and di-sodium phosphates 0.2 M, and borax 0.2 M for pH 4, 7, and 10, respectively). Control experiments with pristine PEDOT:PSS in the absence of any additive were carried out with 10^–4^ to 10^–1^ M aqueous solution of NaCl (Supporting Information Section S7). Raman experiments on metallic substrates were carried out by drop-casting the PEDOT:PSS solutions on gold-coated glasses, zinc, and iron slabs (Alfa Aesar).

Raman data were acquired with a Labram HR-800 spectrophotometer (Horiba/Jobin Yvon) with a He–Ne laser source (λ = 632.8 nm). The laser power was attenuated to 0.3 mW to avoid direct damage of the films. Each measurement was repeated over >10 different regions for each sample. Relative standard deviation of the intensity calculated for the Raman mode of an oxyethylene ring at 583 cm^–1^ was below 5% for homogeneous, nondamaged films.

## 3 Results and Discussion

### 3.1 Working Principle of PEDOT:PSS-Based Raman Probes

**Figure 1:**
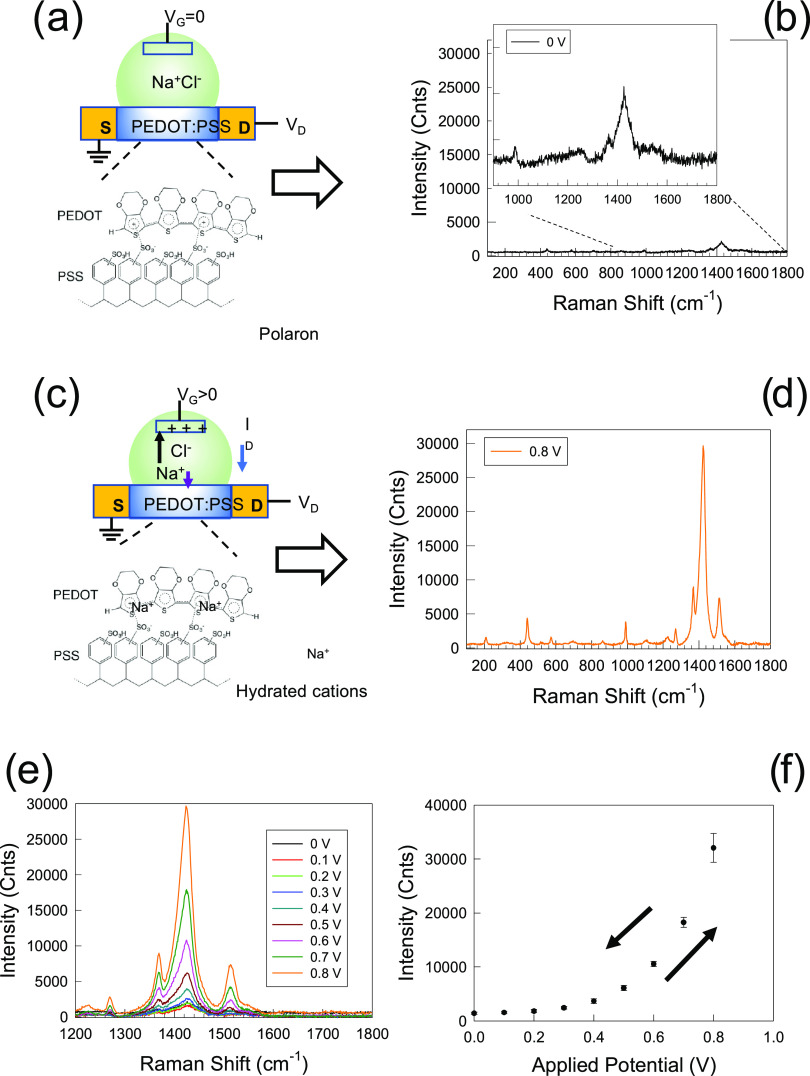
(a, c) PEDOT:PSS at work in a typical organic electrochemical transistor (OECT). Hole-doped PEDOT forms a conducting channel connecting source (S) and drain (D) electrodes. By applying a positive bias (*V*_G_) at the gate electrode, the cations contained in the electrolyte (Na^+^ ions in this example) migrate and penetrate into the PEDOT:PSS network, reducing the number of mobile holes (polarons or bipolarons). As a result, the overall conductivity of the polymer decreases, lowering the current passing between S and D. (b, d) Typical Raman spectra of PEDOT:PSS in doped and dedoped states, respectively. (e) Evolution of the Raman spectrum of PEDOT:PSS as a function of *V*_G_. (f) Increase of the intensity of the PEDOT Raman C_α_=C_β_ symmetric stretching mode as a function of the applied *V*_G_. Within the 0–0.9 V potential range, the Raman response is fully reversible. The error bars of data from 0 to 0.6 V are within the size of the point.

The rationale of Raman sensing based on PEDOT:PSS films is inspired by the working principle of organic electrochemical transistors (OECTs, Figure 1a,c). In OECTs, PEDOT:PSS is normally employed as a p-type semiconducting channel to connect source and drain electrodes. The hole conductivity of PEDOT:PSS is directly controlled by the voltage bias applied at the gate electrode, which drives the cations of the electrolyte to penetrate the polymeric channel, compensating the negatively charged PSS units. As a result, PEDOT passes from a highly conductive oxidized (PEDOT^+^) state to a less conductive neutral (PEDOT^0^) state through the following fully reversible dedoping process
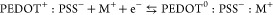
where M^+^ is a generic metal cation of the electrolyte solution, *e.g.*, Na^+^ or K^+^.

Thus, the decrease of source-to-drain current is caused by an overall reduction of the hole concentration, which, in turn, is determined by the gate-controlled injection of cations from the electrolyte. In other words, the electrostatic ion–electronic charges at the PEDOT/electrolyte interface directly modulates the conductivity of PEDOT:PSS, which can be varied more than 3 orders of magnitude by introducing small fractions of ionic charges.^16^

The literature suggests that Raman spectroscopy is a unique, sensitive, and contactless tool for monitoring the PEDOT:PSS transport properties.^13,14^ We assessed this hypothesis by monitoring the evolution of Raman spectra of PEDOT:PSS utilized as a channel in OECTs using a 10 mM NaCl aqueous solution electrolyte (see the Experimental Section and Supporting Information, Section S1 for details). We note that the commercial PEDOT:PSS films cannot be directly utilized in these experiments as PSS rapidly dissolves in milliQ water, leading to phase separation and film decomposition. Thus, the pristine (commercially available) PEDOT:PSS solution was formulated with ethylene glycol (EG) and 3-glycidoxypropyltrimethoxysilane (GOPS, see the Experimental Section for details), and the resulting films were cured at 140 °C for 15 min to promote cross-linking and increase their mechanical stability and adhesiveness to the glass support. The same protocol was utilized for the PEDOT:PSS films tested in all of the experiments reported in the present study.

For clarity, the typical Raman spectrum of an OECT channel based on PEDOT:PSS deposited on glass at a zero voltage bias is shown in Figure 1b, while Figure 1d shows the evolution of the spectrum upon application of a reducing gate voltage *V*_G_ (0.8 V). Figure 1e shows the evolution of Raman spectra when *V*_G_ is swept in the range of 0–0.9 V at steps of 0.1 V. We note that within this voltage range, the spectral changes are fully reversible. The Raman spectrum of PEDOT:PSS contains information about its electronic structure, which is directly influenced by conformational changes of the polythiophene backbone and delocalization of π-electrons. In particular, the spectral region between 1200 and 1600 cm^–1^ displays the most significant changes in intensity, wavenumber, and shape of Raman modes as a function of the applied *V*_G_. On the basis of previous studies on PEDOT and, more generally, polythiophene compounds, these modes are associated to different types of vibrations (Supporting Information Sections S3 and S4).^12,17−19^ The Raman band at the mode at 1260 cm^–1^ originates from C_α_–C_α′_ inter-ring stretching between thiophene units. The peak around 1370 cm^–1^ is due to C_β_–C_β_ and C_α_–C_α′_ stretching, ν_2_ mode (intraring −C_3_–C_4_– and −C_4_=C_5_– in-phase stretching coupled with =CH bending), whereas the main band at about 1430 cm^–1^ is the ν_1_ mode associated to the symmetric stretching of the C_α_=C_β_ bonds of thiophene rings. The asymmetric counterpart can either be enveloped within the width of the ν_1_ mode or exhibit a distinct feature around 1500 cm^–1^. In general, the region between 1500 and 1520 cm^–1^ displays a variable number of Raman modes, whose classification is not well defined, which are usually taken as markers of major structural–electronic changes (π-electron delocalization, electron–phonon coupling, or polaron formation), since they are ascribed to the oscillations of the nuclei along the molecular axis from the ground to the excited state. The intensity of these modes exhibits a progressive, yet nonlinear increase as a function of the bias applied to the gate electrode, as shown in Figure 1f for the symmetric C_α_=C_β_ stretching.

The remarkable change of intensity is caused by several concurrent factors. The progress of reduction is expected to increase the quinoid character of thiophene rings, which extends the delocalization of conjugated π-electrons and makes the molecular structure more rigid. As a result, the optical absorbance of dedoped PEDOT:PSS overlaps with the exciting wavelength of the Raman laser excitation source (λ = 633 nm in the present work), allowing to acquire spectra under resonant conditions, which maximizes the intensity of the Raman modes related to thiophene rings. On the other hand, fully oxidized (*i.e.*, doped) PEDOT:PSS is expected to assume a coil (benzoid) conformation, characterized by an increase of torsional disorder of the molecular backbone, which goes along with decreased rigidity and reduced delocalization of π-electrons. In general, pristine PEDOT:PSS exhibits an intermediate state of oxidation, resulting from the synthesis process, based on the polymerization of EDOT monomers in the presence of Fe^3+^ ions. Thus, it can be either further oxidized or reduced by interaction with oxidizing/reducing species (Supporting Information Section S5).

### 3.2 Using PEDOT:PSS for Probing Redox Mediators: The Role of Saline Buffer

The experiments on OECTs demonstrate that the PEDOT:PSS dedoping/doping process can be monitored in real time by *in situ* microRaman spectroscopy, suggesting the opportunity to exploit the Raman response for probing the redox potential of different classes of analytes under operando-like conditions. For example, Tsai et al. developed an analytical platform for quantitatively detecting H_2_O_2_ and other oxidants from the changes of poly(EDOT–OH) Raman spectra,^13^ while Tan et al. exploited the same concept for indirect detection of glucose, envisioning further extension to the analysis of metabolites and other biological targets.^14^ These studies assume that the Raman spectral features of PEDOT:PSS are only affected by the redox biological events (*e.g.*, glucose oxidation) and not influenced by the electrolyte. Here, we investigate that hypothesis by means of a set of experiments aimed at elucidating the role of ionic electrolytes in mediating transduction of the redox potential of a molecular target. The first series of tests evaluated the possibility to detect the presence and concentration of a biological redox mediator in different aqueous solutions. Glutathione (GSH), one of the most important and abundant antioxidants in biological organisms, was selected as a target. Three different concentrations (viz., 0.5, 5, and 10 mM in milliQ water), corresponding to the typical concentration range of GSH in animal cells, were analyzed. In these tests, each PEDOT:PSS film deposited on glass slides was dipped into the GSH solutions for 30 min and then naturally dried in air at room temperature.

**Figure 2:**
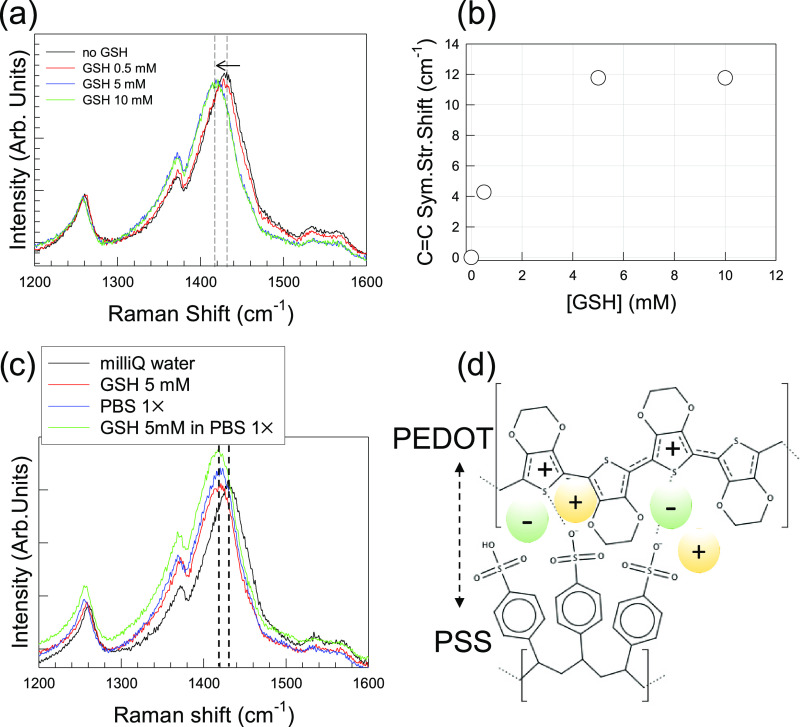
(a) Evolution of Raman spectra of PEDOT:PSS thin films soaked in milliQ water and solutions of GSH at different concentration. (b) Shift of C=C symmetric stretching Raman modes as a function of GSH concentration. (c) Evolution of Raman spectra of PEDOT:PSS thin films soaked in 5 mM solutions of GSH, either in the presence or absence of PBS 1× saline buffer. (d) Scheme representing the penetration of the hydrated electrolyte ions (not to scale), which increases the mutual distance between the PEDOT and PSS chains.

The evolution of the Raman spectra for stabilized PEDOT:PSS films as a function of the GSH concentration is shown in Figure 2a,b. The main band undergoes a progressive, nonlinear downshift as a function of the GSH concentration. In particular, upon soaking in a 0.5 mM solution, the band downshifts of about 4 cm^–1^. When the concentration is incremented by 1 order of magnitude (5 mM), downshifting extends up to 12 cm^–1^, remaining unaltered for 10 mM solutions. Although spectral details revealed a nonlinear response of PEDOT:PSS as a function of the GSH concentration, the systematic downshift of the C=C main modes toward a more extended quinoid conformation and its fully reproducible response (viz., null batch-to-batch variation) might be considered sufficient conditions for detecting GSH at biological concentrations, at least in the 0.5–5 mM range. However, biological fluids, either *in vivo* or *in vitro* conditions, are characterized by the presence of salts. Normally, biochemical and sensing assays extensively utilize saline buffers like phosphate-buffered saline (PBS) solutions. PBS is a mixture of NaCl (137 mM), Na_2_HPO_4_ (10 mM), KH_2_PO_4_ (1.8 mM), and KCl (2.7 mM). Figure 2c shows the spectrum of PEDOT:PSS soaked in a PBS 1× solution. The position of the C=C main band is downshifted to the same value observed for 5 and 10 mM solutions of GSH in milliQ water. This means that a reliable Raman detection of redox-active molecular species under physiological or, more generally, real-world conditions is strongly affected by the presence of salts, which can mask the presence of an analytical target. Salts can have a deep impact on the structure of PEDOT:PSS chains and, therefore, on Raman spectra. Saline ions penetrate the polymeric network, occupying the space between the PEDOT and PSS chains (Figure 2d). In the case of nonoxidizing species, like PBS, ions contribute to the overall charge balancing. In their action, they increase the reciprocal distance between PEDOT and PSS chains. Further complimentary data taken for other individual common electrolytes (KCl, KBr) are reported in Supporting Information Section S6. The addition of GSH to PBS 1× results in a further slight downward shift of about 1 cm^–1^ with respect to either the same concentration in milliQ water or PBS 1×, which demonstrates that even nonionic (*e.g.*, small molecules) species can concur to increasing the separation of PEDOT and PSS chains.

**Figure 3:**
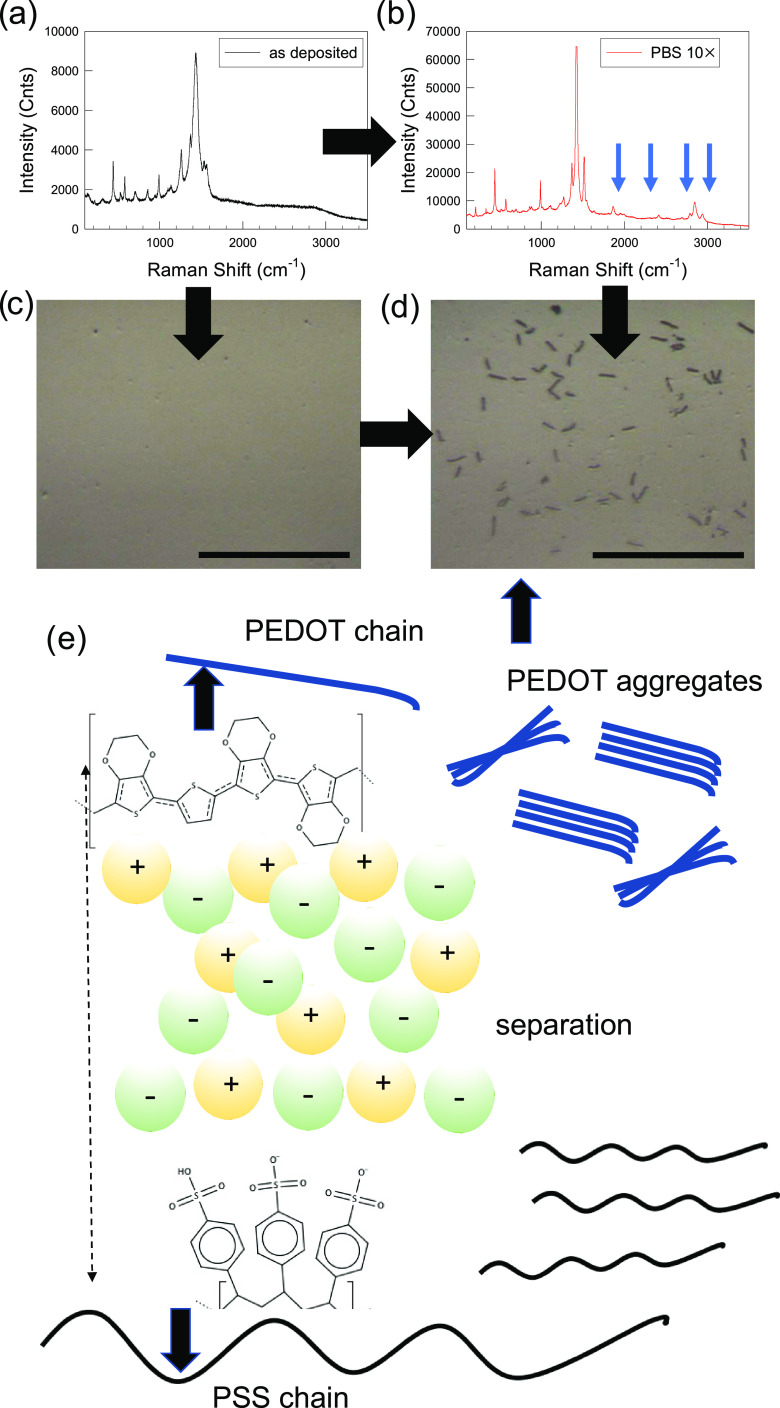
Evolution of the PEDOT:PSS Raman spectrum soaked in (a) milliQ water and (b) PBS 10× solutions. The blue arrows indicate the new bands that appear upon extended separation of PEDOT and PSS units. (c) Optical microscope image of PEDOT films before and (d) after soaking in PBS 10× solution. In the latter case, microrods formed upon PBS 10×-induced phase separation are visible. Scale bars: 20 μm. (e) Cartoon with a scheme of separation between PEDOT and PSS chains upon soaking in PBS 10×. Charged spheres represent hydrated ions from the buffer saline electrolyte (image not to scale).

To further investigate the role of electrolyte in promoting chain separation, the concentration of PBS was increased by 1 order of magnitude (from 1 to 10×). This means that the concentration of NaCl raised to 1.37 M. As shown in Figure 3, the Raman spectrum changes remarkably before and after soaking the PEDOT:PSS films in PBS 10×. The Raman modes become narrower and more intense (enhancement factor > 7× for the symmetric stretching C=C main band), following the same trend observed for OECTs operated at large positive *V*_G_, as shown in Figure 1. The extension of the spectrum up to 3500 cm^–1^ allows us to note the appearance of modes in the region of −CH_2_– stretching signals (2900–3000 cm^–1^), as well as others around 1800–1900 cm^–1^, which are absent in the Raman spectra of PEDOT. Although a detailed investigation of their origin is beyond the scope of this work and most of Raman data on PEDOT:PSS are normally limited within the 0–1700 cm^–1^ range, a comparison with the few studies dedicated to the redox switching of Raman spectra reveals that these modes do not appear in the absence of PSS.^13^ The *in situ* characterization under an optical microscope upon thorough washing in ultrapure (milliQ) water reveals evidence of demixing between PEDOT and PSS chains, resulting in the formation of PEDOT rod-type macroaggregates that are not present in the pristine PEDOT:PSS (Figure 3c,d). Similar features were reported in the literature for differently treated PEDOT:PSS samples, yet only at the nanoscale.^20,21^ Atomic force microscopy (AFM) and conductive scanning probe microscopy (C-SPM) reveal that the macroaggregates are not uniform with thicker regions that are significantly less conductive than the planar background (Supporting Information Sections S8–S10). Interestingly, the Raman spectrum after extended washing shows the same spectrum of the pristine PEDOT:PSS film. This suggests that the extra-Raman modes observed in samples treated in PBS 10× might be characteristic of PSS segregation or reflect the increased crystallization of the PEDOT chains during chain separation/segregation. This framework is consistent with the small-angle X-ray scattering (SAXS)- and small-angle neutron scattering (SANS)-based study on the interaction of ionic liquid electrolytes with PEDOT:PSS, recently reported by Li et al., which demonstrated that high ionic concentrations destabilize the interaction between the chains, leading to their excessive separation and segregation.^22^ In particular, this work showed a progressive transition from a core/shell to rodlike structures as a function of the electrolyte concentration. Partial separation between PEDOT and PSS chains was observed for the concentration of electrolyte around 0.09 M, whereas an increase of the concentration to 0.33 M results in excessive separation of the chains, which gives rise to phase segregation.^22,23^

### 3.3 Role of Substrate

**Figure 4:**
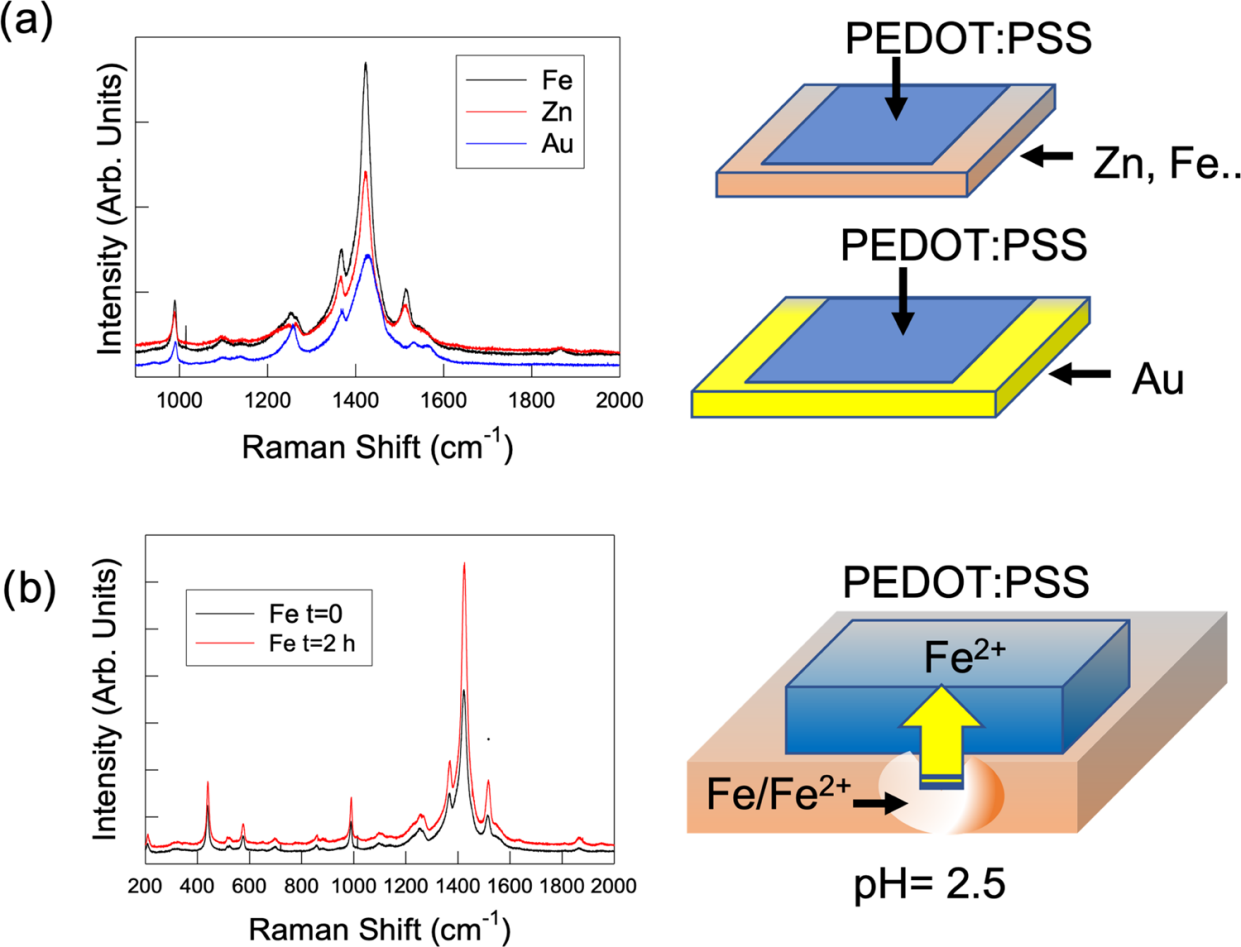
(a) Raman spectra of PEDOT:PSS films deposited on different metals. (b) Evolution of the Raman spectrum of films deposited onto Fe substrates as a function of time (see the main text for details).

Interestingly, the same effect was also observed by direct deposition of PEDOT:PSS thin films on different types of metallic substrates (Figure 4a). We note that PEDOT:PSS exhibits a Raman spectrum analogous to those observed in samples treated in PBS 10× solutions or electrically biased when deposited onto iron or zinc foils (or powders), whereas it remains almost unaltered in contact with gold. Although at first sight, this behavior could be interpreted by analyzing the respective work functions of metal and PEDOT, the changes in Raman spectra results from the structural and electronic modifications of PEDOT:PSS, which are always mediated by ionic diffusion. In the case of zinc and iron substrates, the ions come from direct dissolution induced by low pH. In fact, we note that the pH of PEDOT:PSS pristine solution is 2.5 and acidic conditions drive metal surface etching. As a result, Zn^2+^ and Fe^2+^ are released from metallic substrates and penetrate the PEDOT:PSS network with the same effects produced by other saline ions. The fact that the iron substrate does not exhibit the feature typical of oxidation suggests that iron is leached away from the substrate in the form of less oxidizing Fe^2+^ ions. On the other hand, gold is not etched under these conditions and Raman spectra are analogous to those obtained from glass substrates. A further proof of the process dynamics was achieved by acquiring the Raman spectrum on the same region after two hours from the first measurement (Figure 4b). In this case, the intensity of the Raman spectrum is significantly increased. This allows us to exclude direct charge transfer from metal to PEDOT, which should be very fast, as a direct cause of the spectral evolution. On the other hand, time increases the PEDOT:PSS uptake of ions dissolved from the substrate, which results in Raman enhancement.

### 3.4 Role of pH

**Figure 5:**
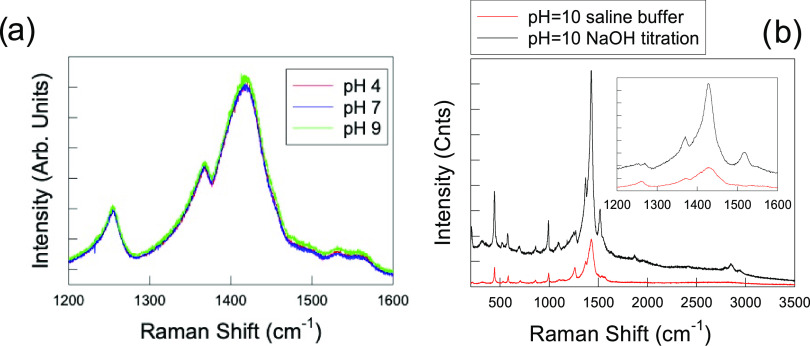
(a) Raman spectra of PEDOT:PSS films in standard saline buffers at three different pH values. (b) Raman spectrum of a film soaked in NaOH solution at pH = 10, showing the effects of PSS deprotonation compared to a film immersed in saline buffer solution at the same pH.

In 2004, de Kok et al.^24^ reported an experimental study showing that the same Raman features associated to the application of a reducing voltage bias can be obtained simply by shifting the pH of PEDOT:PSS toward alkaline conditions. In particular, they carried out a chemical titration by adding increasing amounts of NaOH to a solution containing PEDOT:PSS thin films. The evolution of the Raman spectra of the films was identical to that observed by applying a reducing bias, reaching the same features of a complete reduction (−1.2 V) at pH = 9.9. However, the fact that the Raman spectrum is also identical to that observed in our experiments with highly concentrated saline buffers (*e.g.*, PBS 10×, Figure 4b) suggests that phase separation between PEDOT and PSS could be at a basis of the pH-dependence reported in the literature. To verify that hypothesis, we soaked the PEDOT:PSS films in aqueous solutions buffered with salts at three different pH values, 4, 7, and 10. Figure 5a shows that the all of the Raman spectra were identical, irrespective of different values of pH, demonstrating that pH is not *per se* a factor that influences the Raman response of PEDOT:PSS. In our experiment, pH was set by means of saline buffers with an overall concentration comparable to that of PBS 1×, which introduces ions, yet maintaining the stability of the PEDOT:PSS interface. This mismatch between our results and those reported by de Kok et al. can be explained by considering that in its pristine form, the pH of PEDOT:PSS is 2.5. In these conditions, most of the PSS chains are indeed protonated.^25^ de Kok et al. regulated pH by direct addition of NaOH.^24^ In this way, they induced a progressive deprotonation of the PSS chains. As a result, the PEDOT:PSS interface is destabilized until the onset of ionomer separation. Thus, the final Raman spectrum represents a snapshot of the phase-separated material, not the effect of different pH. To further prove this hypothesis, we experimentally confirmed that the same results obtained by de Kok et al. can be achieved by adding NaOH to our films (Figure 5b).

### 3.5 Oxidizing Conditions

Overall, our experiments demonstrated that the cause of remarkable modifications of the Raman signals observed upon application of a reducing voltage bias is indeed associated to the structural modification of the polymeric network driven by ion and/or small molecule insertion. This means that the same effect on the Raman output can be produced from different inputs (applied voltage, highly concentrated salts solutions, *etc*.), provided that PEDOT and PSS chains are well separated and sufficiently rigid. Thus, in the presence of nonoxidizing electrolytes, the Raman spectra of PEDOT:PSS can only provide direct information of the concentration of ions and their effect on the structure of the polymeric network; however, they are not suitable for evaluating the redox potential of reducing species in a reliable and quantitative way.

**Figure 6:**
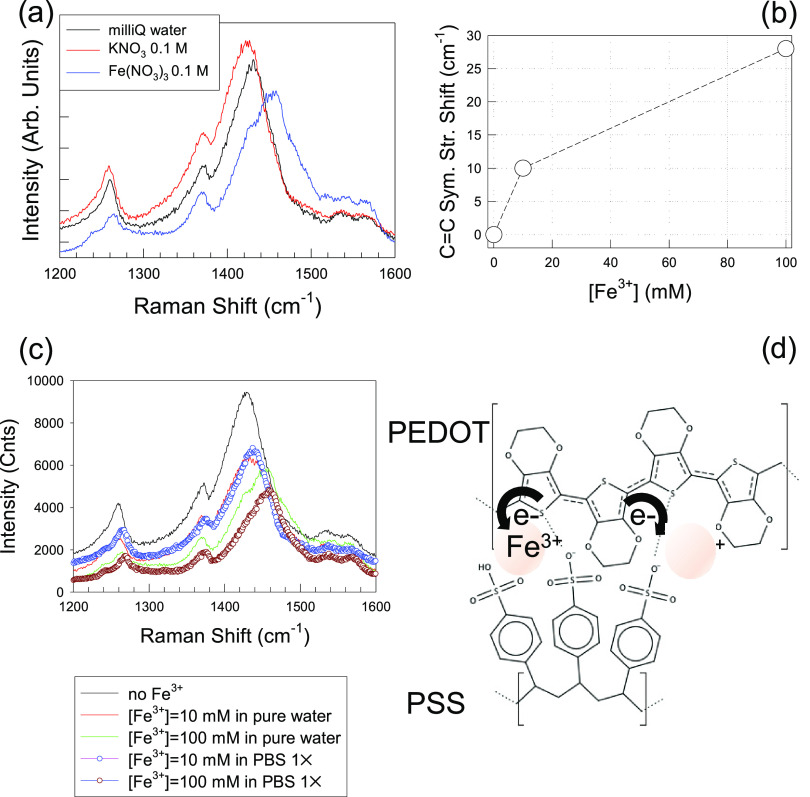
(a) Raman spectra of PEDOT:PSS films in KNO_3_ and Fe(NO_3_)_3_ solutions at the same concentration (100 mM). (b) Shift of C=C symmetric stretching of Raman modes at 10 and 100 mM. (c) Evolution of Raman spectra of PEDOT:PSS immersed in Fe^3+^ solutions in either ultrapure (milliQ) water or PBS 1×. (d) Scheme of Fe^3+^-induced oxidation of the PEDOT chains.

On the other hand, electrolyte and molecular species can also cause the oxidation of PEDOT by directly drawing electrons from thiophene rings. Fe^3+^ ions are an example of oxidizing species that are typically utilized to dope PEDOT:PSS (Figure 6d). As shown in Figure 6a, oxidation by Fe(NO_3_)_3_ results in upshifting and broadening of the C=C symmetric stretching modes, which systematically decrease their intensity. The nitrate counterions do not have any significant effects on the appearance of the Raman spectrum as demonstrated by control experiments in nonoxidizing KNO_3_ at the same concentration, which shows the same Raman response (downshift, slight increase in intensity) observed for other nonoxidizing salts. Upshifting depends on the concentration of the Fe^3+^ salt reaching 10 and 28 cm^–1^ in the case of 10 and 100 mM solutions, respectively (Figure 6b). Interestingly, experiments carried out in the presence of PBS 1× show that the Raman response is qualitatively the same as that observed for Fe^3+^ solutions in ultrapure water (Figure 6c). This means that not only the saline buffer does not interfere with oxidation but it also supports charge transfer in highly concentrated oxidant solutions. This conclusion is supported by the extended decrease in the Raman intensity of C=C symmetric stretching band and its further slight upshifting in comparison to the pure-water counterpart for the 100 mM samples.

### 3.6 Summary

**Figure 7:**
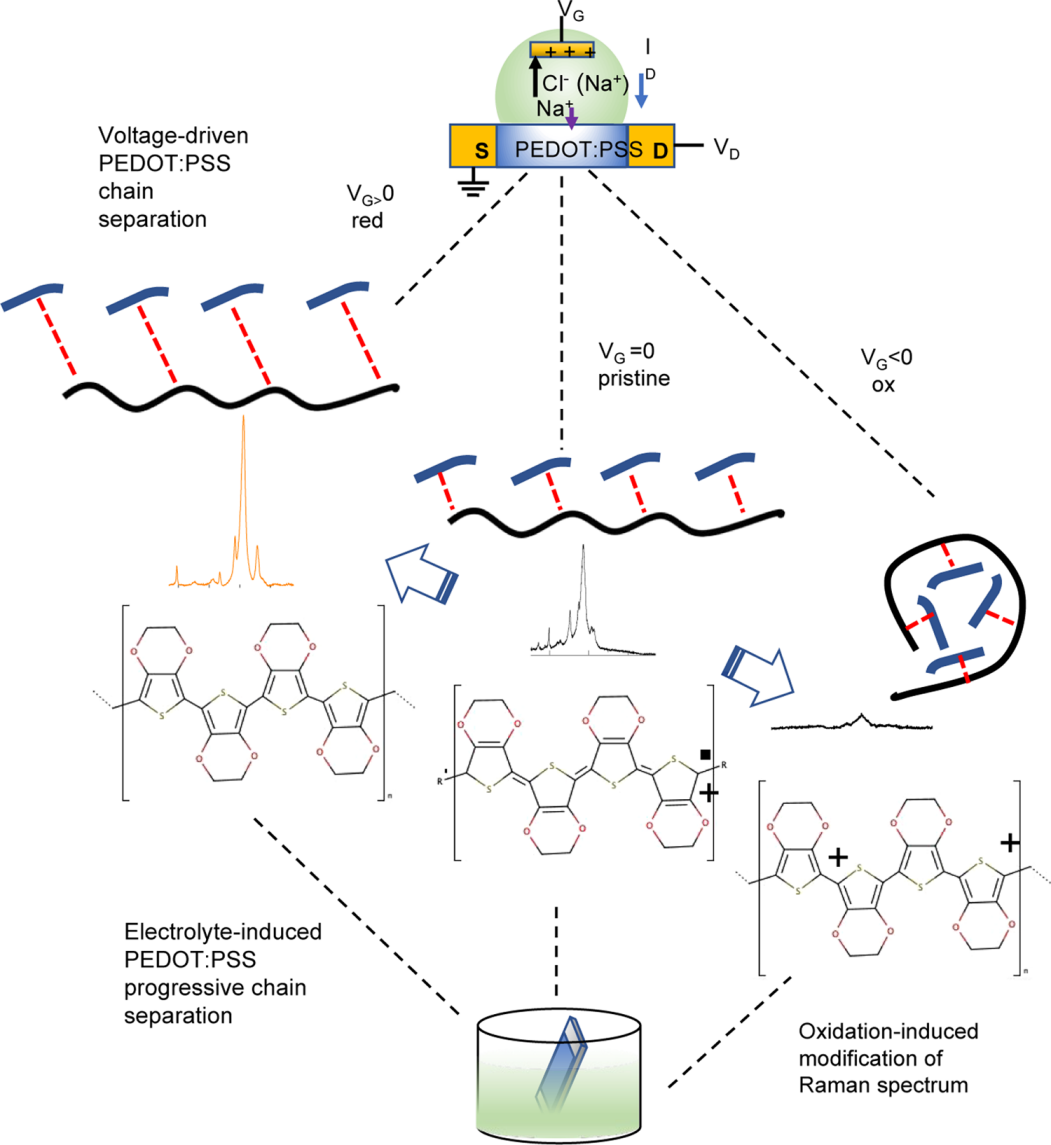
Cartoon showing a comparative summary of the main results of Raman experiments in both OECTs and simple PEDOT:PSS films.

The scheme reported in Figure 7 summarizes the main findings of this study, comparing the different uses of Raman spectra of PEDOT:PSS for monitoring the electric potential of a solution containing a target molecule in the case of either OECT-based devices or simple planar thin-film chips, as those investigated here. In OECTs, the Raman spectrum reproduces with high reliability, the voltage bias applied at the gate electrode, for both oxidizing and reducing potential sweeps. Such a high-fidelity response is driven by the architecture of OECTs. In fact, the concentration of saline buffer is usually within 10–100 mM, which prevents irreversible phase separation between the chains PEDOT and PSS, and the diffusion of ions across the polymeric network is precisely controlled by the gate voltage. Moreover, the presence of source and drain electrodes acts as sinks for the polymeric charges, preventing their accumulation and irreversible chain separation. On the other hand, in thin-film chips, we observed two different behaviors. Oxidizing ionic species, like Fe^3+^, enable direct oxidation of the PEDOT units. Here, the presence of other nonoxidizing electrolytes, like saline buffers, does not interfere in the Raman detection of charge transfer as the interaction between PEDOT and PSS units is directly dictated by the increase of positive charges on the PEDOT chains upon oxidation. On the other hand, the effect of reducing species is strongly affected by salts, which penetrate the PEDOT:PSS network, inducing a progressive separation of the two ionomers. The same effect can be achieved also by deprotonation of PSS chains, as demonstrated in experiments on pH-dependence. When the concentration of the electrolyte is particularly high (*e.g.*, as in the case of PBS 10×), chain separation is irreversibly extended, leading to the formation of PEDOT crystalline aggregates, as previously shown in Figure 3.

## 4 Conclusions

This study aimed at assessing the rationale of Raman sensing mediated by PEDOT:PSS films, which is based on the assumption that chemical events involving charge transfer in solution can be reliably transduced as specific variations of the vibrational spectrum of PEDOT:PSS itself without the interference of any electrolyte. However, the experimental results show that, *de facto*, Raman spectra reflect the structural conditions of the PEDOT:PSS network and their modifications are mainly determined by the interactions between the chains of the two ionomers. In particular, the same “reduction” signals observed upon the application of a gate potential can also be obtained through a drastic variation of salt concentration or by introducing any other factor that increases the distance between PEDOT and PSS and modifies the rigidity and structural order of PEDOT units. The transmission of information related to the electrochemical potential of target medium does not occur through direct charge transfer, but it is mediated by diffusion of hydrated ions (or small molecules, as in the case of glutathione). Therefore, Raman spectra do not provide data that can be directly correlated with the redox state of a real system (*e.g.*, a biological fluid) containing nonoxidizing ions and other possible interferents in a high concentration range (10^–1^ to 10^–3^ M). At the same time, ionic concentrations that are too low (10^–4^ M) are not sufficient to induce a significant modification of the interactions between PEDOT and PSS, which limits the sensitivity of Raman sensing. On the other hand, the presence of oxidizing ions, like Fe^3+^, can be evaluated straightforwardly even in the presence of saline buffers. This work sheds light on past literature data about PEDOT:PSS-based Raman sensing and their interpretation, bringing a new theoretical support to design future experiments. This study also remarks the usefulness of Raman spectroscopy to characterize the structure of PEDOT:PSS films^26−28^ at the atomic level and investigate the dynamics of mixed electronic/ionic charge transfer in three-dimensional conductive polymers.^29^ It may also provide an indirect, yet sensitive way to quantify the overall concentration of electrolytes and small molecules in different environments, such as biological fluids or water, in real time. In this context, PEDOT:PSS films offer a cheap, biocompatible, flexible, and Raman-responsive tool for the fabrication of point-of-use, all-optical sensors.

## Supporting Information Available

Details of the setup for Raman experiments on PEDOT:PSS-based OECTs (Section S1); AFM measurement of thin films; average thickness (Section S2); benzoid and quinoid structure of PEDOT (Section S3); Raman spectra of PEDOT:PSS; literature-based assignment of the main bands (Section S4); scheme of the oxidation states of PEDOT (Section S5); Raman spectra of PEDOT:PSS in nonoxidizing halide salts (Section S6); Raman spectra of PEDOT:PSS without additives in NaCl solutions (Section S7); AFM image of a dimer of PEDOT macroaggregates (Section S8); AFM image of a PEDOT macroaggregate (Section S9); and conductive scanning probe microscopy of the PEDOT macroaggregate shown in Section S9 (Section S10) (PDF)
