# Supplementary material for: Why PEDOT:PSS Should Not Be Used for Raman Sensing of Redox States (and How It Could Be)
Source: ACS Appl Mater Interfaces. 2022 Dec 7;14(50):56363–73. doi: 10.1021/acsami.2c17147 (PMC9782336; doi:10.1021/acsami.2c17147)
Supplement: Supplementary file 1 — am2c17147_si_001.pdf [file am2c17147_si_001.pdf]

# SUPPORTING INFORMATION

# Why PEDOT:PSS Should Not Be Used for Raman Sensing of Redox States (and How It Could Be)

*Ivano Alessandri*,[1,2,3]Fabrizio Torricelli,[1] Beatrice Cerea,[1] Michele Speziani,[1] Paolo Romele,[1] Zsolt Miklos Kovacs-Vajna,[1] Irene Vassalini,[1,2,3]*

[1]Department of Information Engineering, University of Brescia, via Branze 38, 25123 Brescia (Italy)

[2] INSTM-National Consortium for Materials Science and Technology, UdR Brescia, via Branze 38, 25123 Brescia (Italy)

[3] CNR-INO, UdR Brescia, via Branze 38, 25123 Brescia (Italy)

**Corresponding Author**

***Ivano Alessandri, e-mail: ivano.alessandri@unibs.it**

# Content

**S1.** Details of setup for Raman experiments on PEDOT:PSS-based OECTs

**S2.** AFM measurement of thin films. Average thickness.

**S3.** Benzoid and quinoid structure of PEDOT

**S4.** Raman spectra of PEDOT:PSS. Literature-based assignment of the main bands

**S5.** Scheme of the oxidation states of PEDOT

**S6.** Raman spectra of PEDOT:PSS in non-oxidizing halide salts

**S7**. Raman spectra of PEDOT:PSS without additives in NaCl solutions

**S8.** AFM image of a dimer of PEDOT macroaggregates

**S9.** AFM image of a PEDOT macroaggregate

**S10.** Conductive scanning probe microscopy of the PEDOT macroaggregate shown in S9.

## S1. Details of setup for Raman experiments on PEDOT:PSS-based OECTs

The analysis of variation of the Raman spectrum of PEDOT:PSS films utilized as channels for OECTs was carried out by means of a home-made fluidic cell (Figure S1) loaded with a NaCl solution (concentration: 10 mM), connected with a potentiostat. The Vg was swept from 0 to 0.9 V at step of 0.1 V, with the source electrode grounded and the drain electrode polarized at a constant potential.

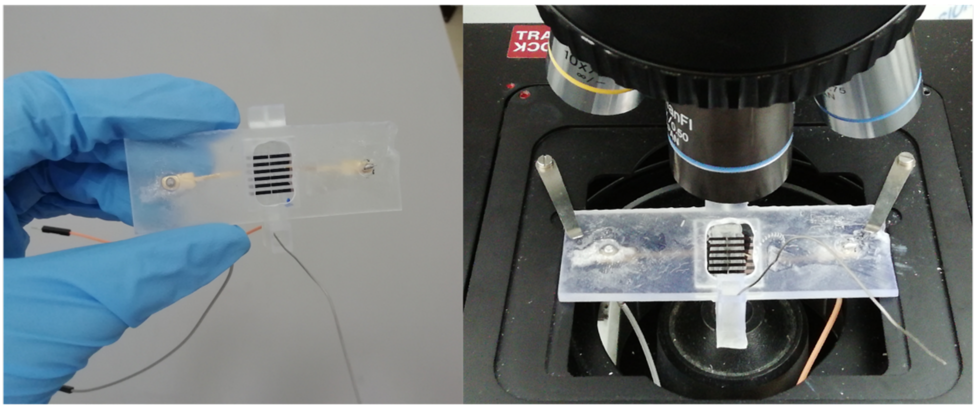

**Figure S1.** a) Pictures of the fluidic cells containing the PEDOT:PSS-based OECTs, fabricated with the same protocol described in reference 10 (main text), using source and drain electrodes made of gold and Ag/AgCl pellet electrode for the gate. The gate length was 100 μm.

# S2. AFM measurement of thin films. Average thickness.

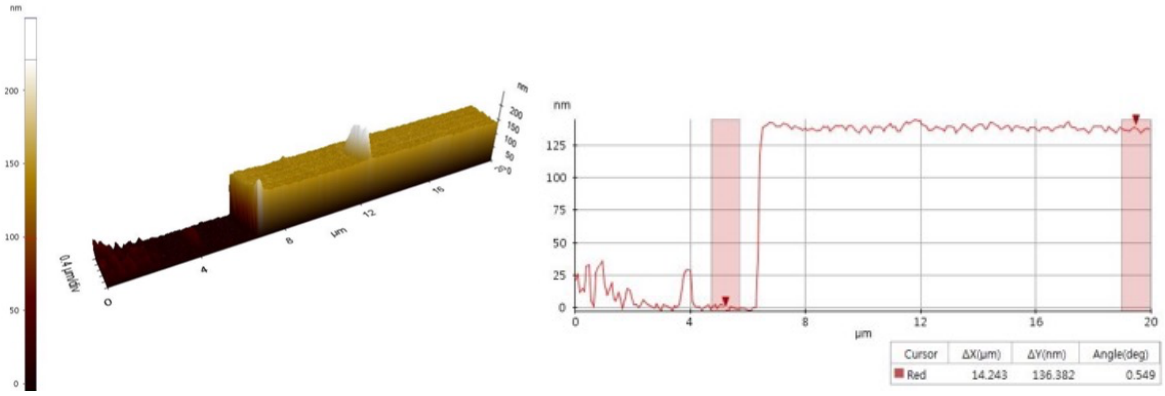


**Figure S2.** Average thickness (136.4 nm) of the PEDOT:PSS measured by atomic force microscopy (AFM, Park Systems Corp. NX10 ).

**S3. PEDOT: Benzoid and quinoid structures**

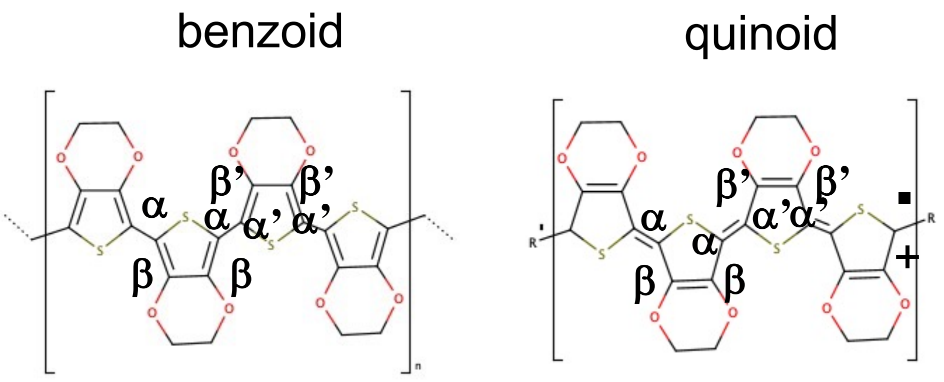


**Figure S3.** Benzoid and quinoid structures of PEDOT.

**S4. Raman spectra of PEDOT:PSS. Literature-based assignment of the main bands.**

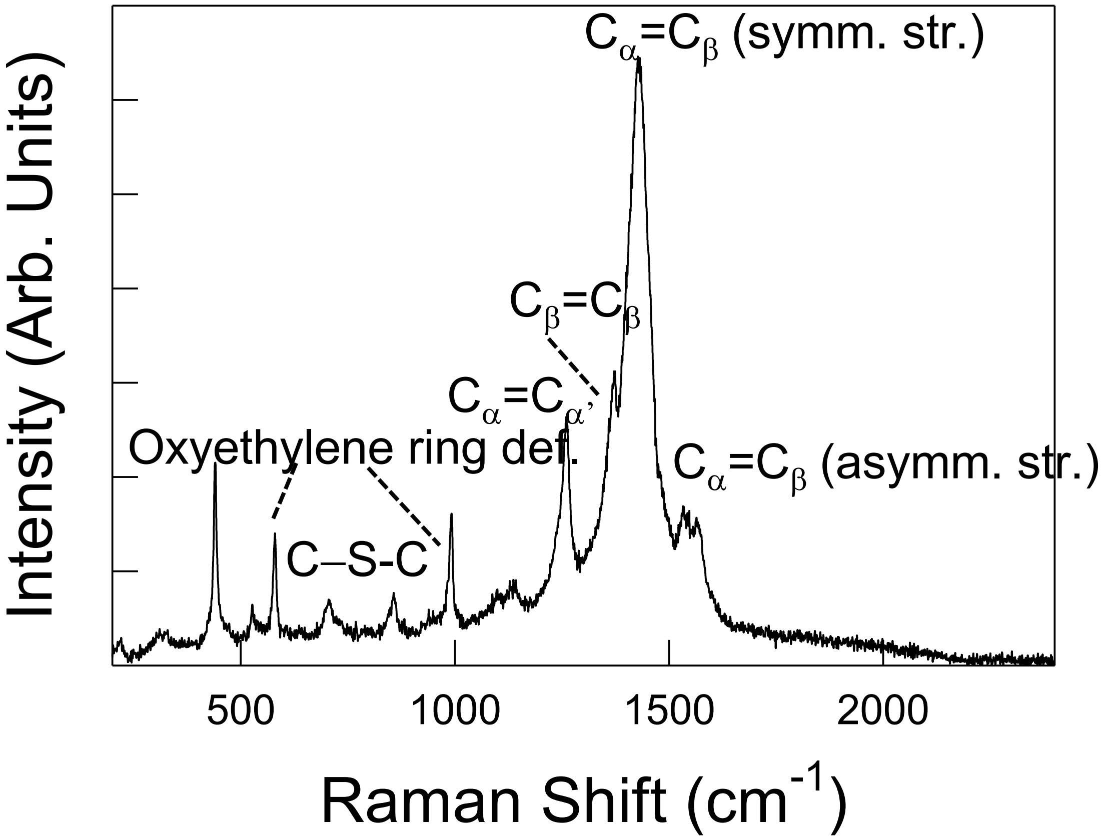


**Figure S4.** Raman spectrum of a pristine film of PEDOT:PSS and assignment of the main bands, based on ref.12 of the main text.

**S5. Scheme of the oxidation states of PEDOT**

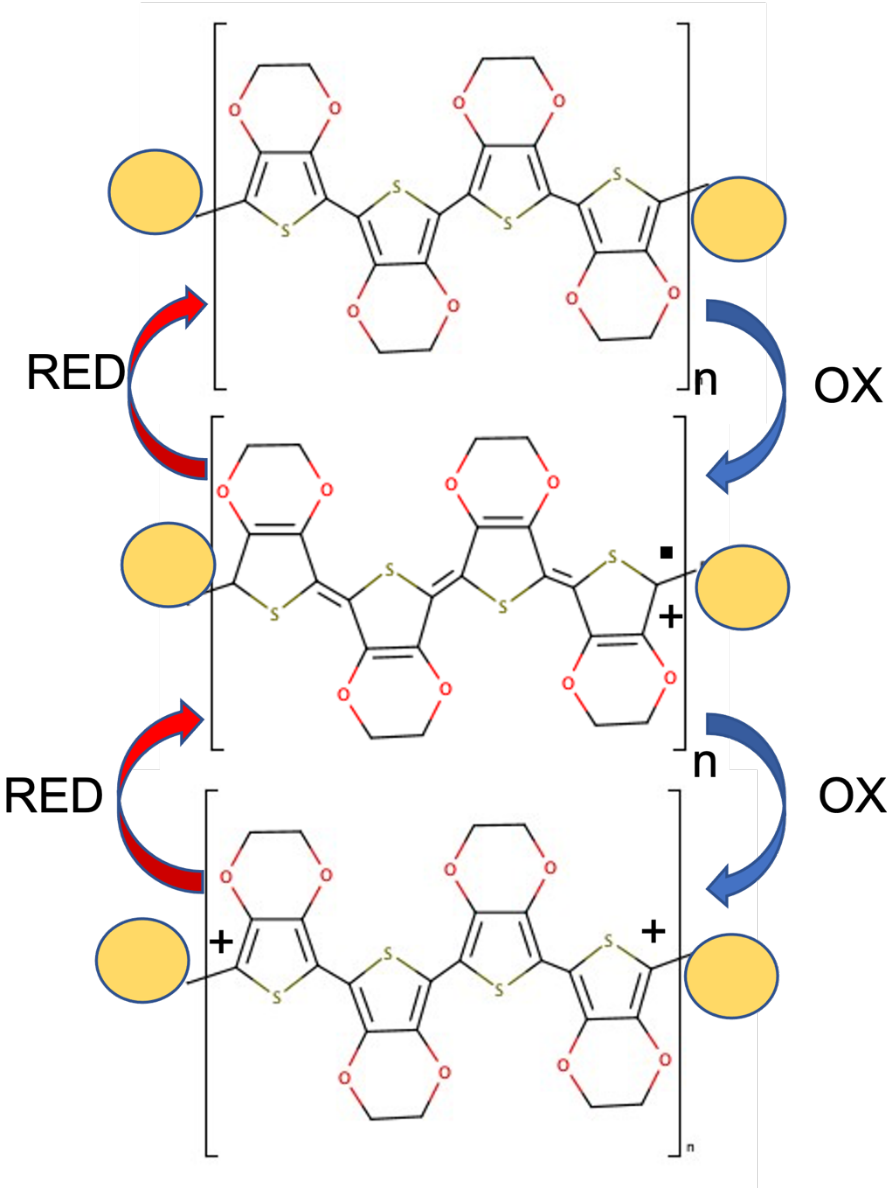


**Figure S5.** Scheme of the oxidation states of PEDOT:PSS. Pristine PEDOT:PSS corresponds to the intermediate, polaronic state.

## S6. Raman spectra of PEDOT:PSS in non-oxidizing halide salts

Figure S4 shows the modifications in intensity and spectral position of the Raman main bands of PEDOT:PSS films previously soaked in different saline solutions, characterized by the same type of cations ($K^+$) and different anions ($Cl^-$, $Br^-$). When the concentration of the saline solution is the same, we note that the band shift is similar for all the spectra, irrespective of the specific type of salt. It is important to remark that the C=C band downshift is associated to the insertion of ions or small molecules between the PEDOT and PSS chains and it is not representative of the redox potential of the environment under investigation.

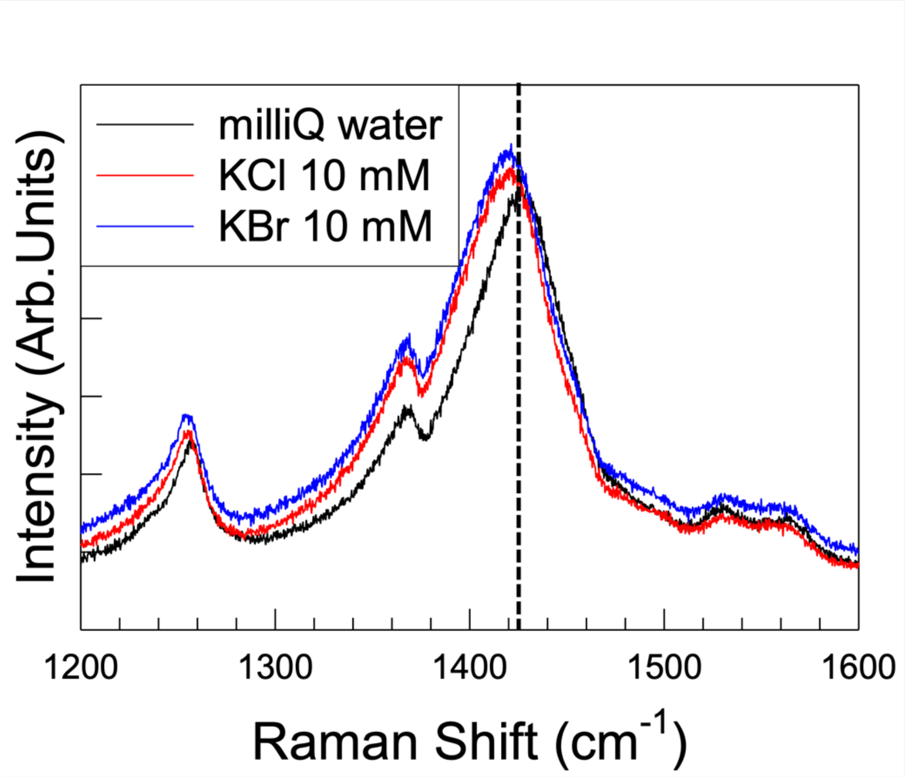


**Figure S6.** Evolution of the Raman spectrum of PEDOT:PSS films soaked in 10 mM solutions of KCl and KBr.

**S7. Raman spectra of pristine PEDOT:PSS without any additive in NaCl solutions**

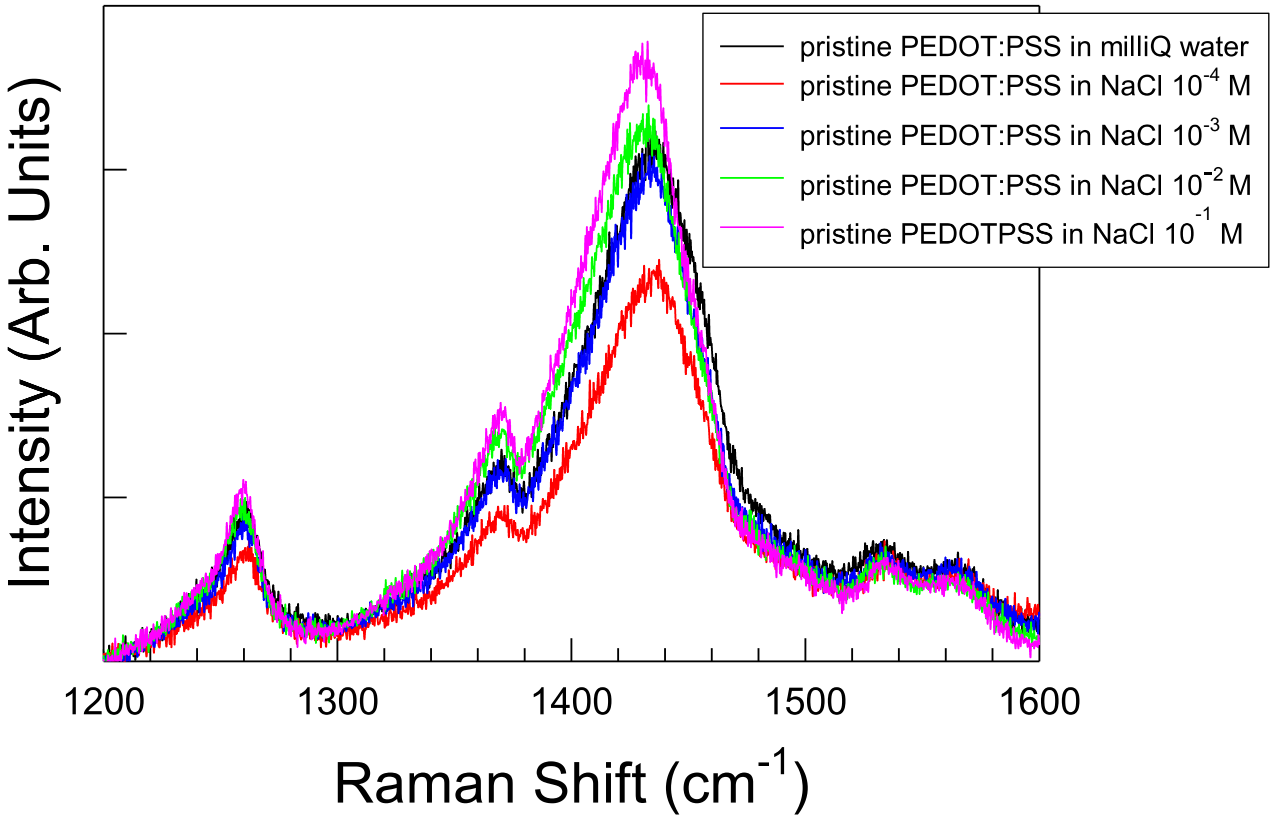


**Figure S7.** Evolution of the Raman spectrum of PEDOT:PSS films without addition of any additive, soaked in solutions of NaCl at different concentrations.

## S8. AFM image of a dimer of PEDOT macroaggregates

Selected macroaggregates were characterized by atomic force microscopy and conductive scanning probe microscopy (Instrument: Park Systems Corp. NX10).

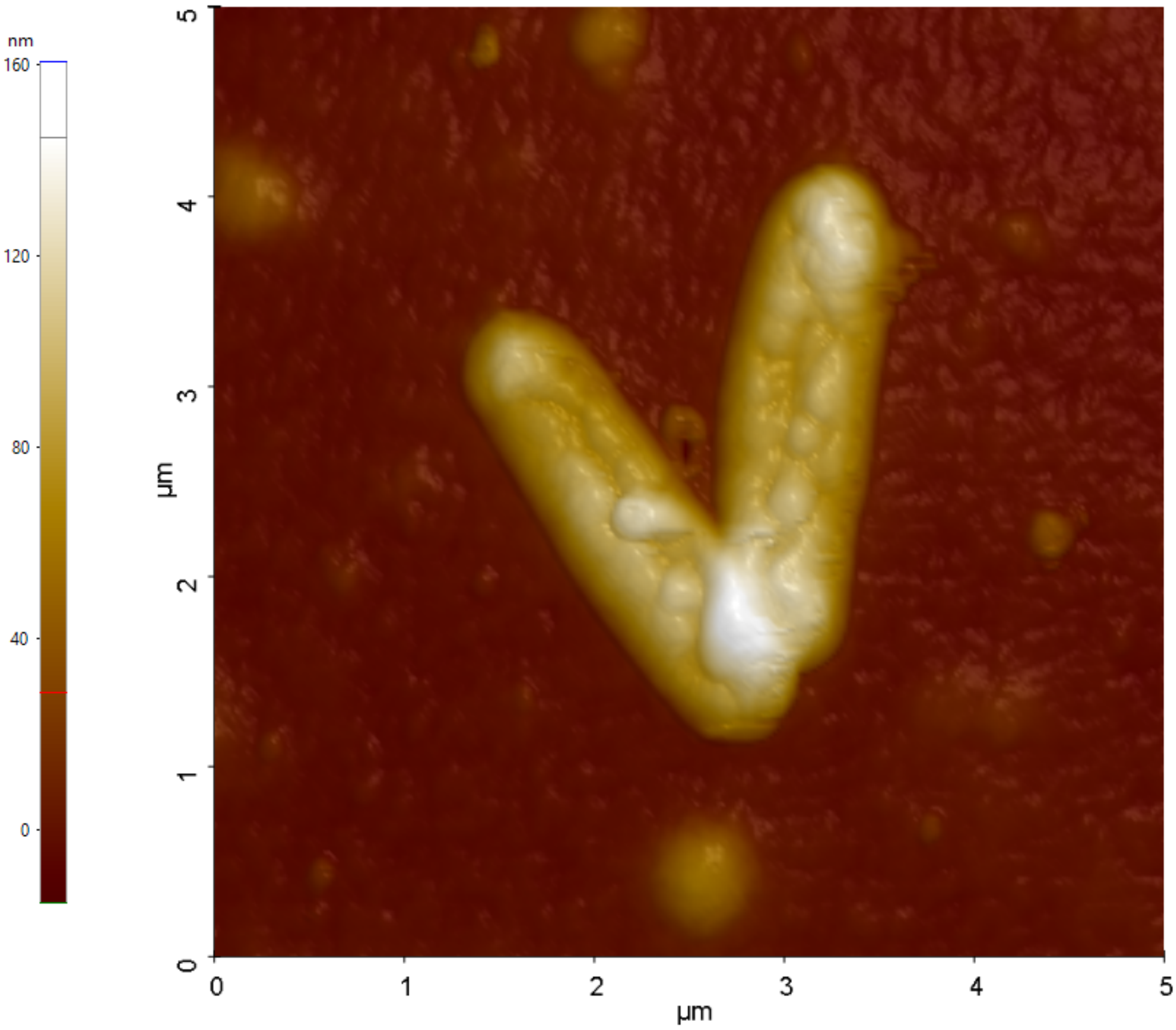


**Figure S8**. AFM image showing a dimer of PEDOT macroaggregates. The tip used is an OMCL-AC160TS by Olympus made of N-doped silicon with typical 7nm radius and 26 N/m Force Constant at its first resonant frequency around 300kHz in AC mode.

## S9. AFM image of a PEDOT macroaggregate

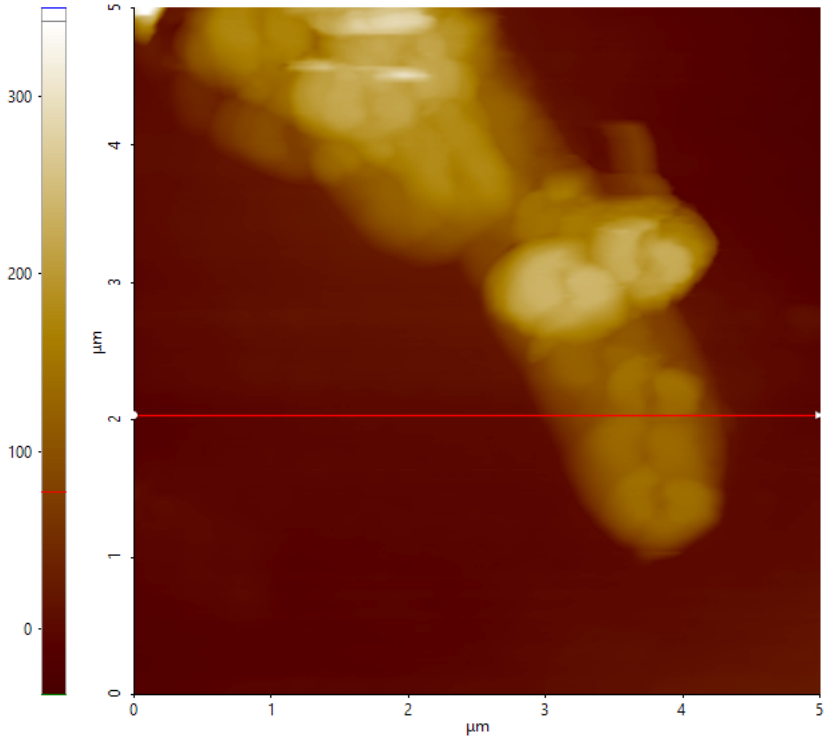


**Figure S9.** Atomic Force Microscopy of a PEDOT macroaggregate.

**S10. Conductive scanning probe microscopy of the PEDOT macroaggregate shown in S9**

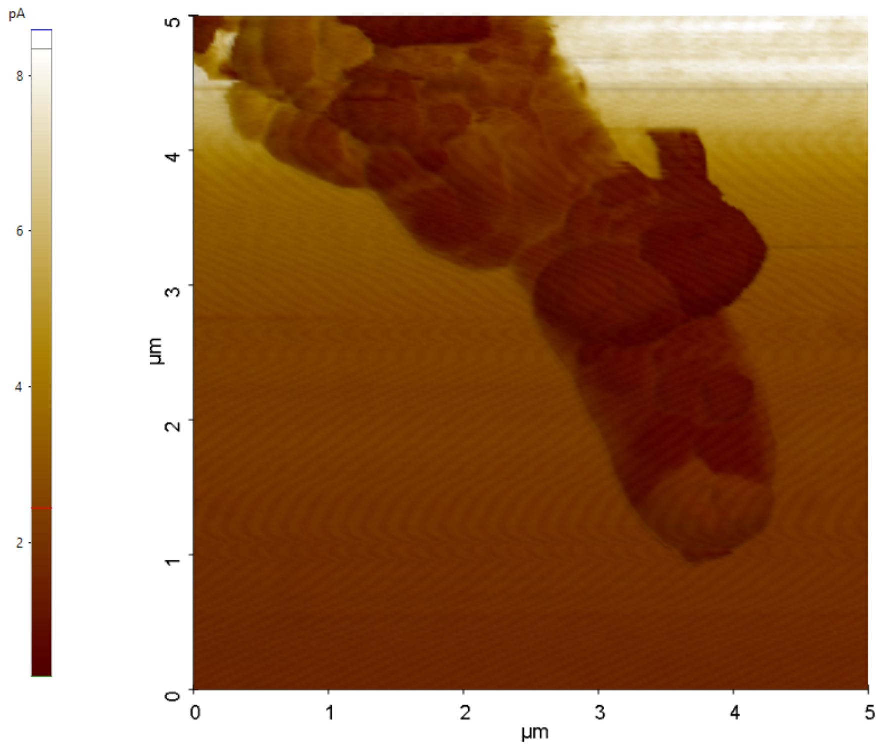


**Figure S10.** Conductive Probe Microscopy of the PEDOT macroaggregate shown in Figure S9. The conductive background is contributed by the planar PEDOT:PSS film. The tip used is an uncoated Point Probe Plus CONTSCR by Nanosensors made of highly doped silicon with typical 7nm radius and 0.2 N/m Force Constant. The current was measured in contact mode using an external VECA (Variable Gain Current Amplifier) amplifier with $10^9$ gain.
